# Slowed Saccadic Reaction Times in Seemingly Normal Parts of Glaucomatous Visual Fields

**DOI:** 10.3389/fmed.2021.679297

**Published:** 2021-08-26

**Authors:** Gijs Thepass, Hans G. Lemij, Koenraad A. Vermeer, Johannes van der Steen, Johan J. M. Pel

**Affiliations:** ^1^Vestibular and Ocular Motor Research Group, Department of Neuroscience, Erasmus MC, Rotterdam, Netherlands; ^2^Rotterdam Ophthalmic Institute, Rotterdam, Netherlands; ^3^Glaucoma Service, Rotterdam Eye Hospital, Rotterdam, Netherlands; ^4^Royal Dutch Visio, Huizen, Netherlands

**Keywords:** eye movement perimetry, saccadic reaction time, eyetracking, visual field, glaucoma

## Abstract

**Purpose:** In eye movement perimetry, peripheral stimuli are confirmed by goal-directed eye movements toward the stimulus. The saccadic reaction time (SRT) is regarded as an index of visual field responsiveness, whereas in standard automated perimetry (SAP), the visual field sensitivity is tested. We investigated the relation between visual field sensitivity and responsiveness in corresponding locations of the visual field in healthy controls and in patients with mild, moderate and advanced glaucoma.

**Materials and Methods:** Thirty-four healthy control subjects and 42 glaucoma patients underwent a 54-point protocol in eye movement perimetry (EMP) and a 24-2 SITA standard protocol in a Humphrey Field Analyzer. The visual field points were stratified by total deviation sensitivity loss in SAP into 6 strata. A generalized linear mixed model was applied to determine the influence of the various factors.

**Results:** The generalized linear mixed model showed that the mean SRT increased with increasing glaucoma severity, from 479 ms in the control eyes to 678 ms in the eyes of patients with advanced glaucoma (*p* < 0.001). Mean SRTs significantly increased with increasing SAP sensitivity loss. Even at the locations where no sensitivity loss was detected by SAP (total deviation values greater or equal than 0 dB), we found lengthened SRTs in mild, moderate and advanced glaucoma compared to healthy controls (*p* < 0.05) and in moderate and advanced glaucoma compared to mild glaucoma (*p* < 0.05). At locations with total deviation values between 0 and −3 dB, −3 and −6 dB and −6 and −12 dB, we found similar differences.

**Conclusions:** The lengthened SRT in areas with normal retinal sensitivities in glaucomatous eyes, i.e., planning and execution of saccades to specific locations, precede altered sensory perception as assessed with SAP. Better understanding of altered sensory processing in glaucoma might allow earlier diagnosis of emerging glaucoma.

## Introduction

In patients with suspected or confirmed glaucoma, visual field (VF) testing is the standard procedure to assess visual field sensitivity (VFS). In glaucoma degeneration of ganglion cells and their axons, which form the optic nerve, leads to permanent loss of the associated parts of the visual field (1). The two main types of glaucoma are open-angle glaucoma and angle-closure glaucoma. The exact etiology is unknown but the most important risk factor is high intraocular pressure. An important third type is secondary glaucoma which is the result of an identifiable cause. Eventually, the loss of ganglion cells results in visual impairment due to tunnel vision and can result in blindness. The prevalence for the population 40–80 years is 3.54% therefore 76 million are affected in 2020 increasing to 112 million in 2040 (2). Treatment that lowers the intra ocular pressure slows progression but it is not curative. Standard automated perimetry (SAP), which has been widely used for several decades, is currently the gold standard in functional testing (3). During SAP, the subject's responses to visual stimuli projected in the central and peripheral visual field are evaluated while the subject is instructed to maintain fixation on a central target. Despite its worldwide application, the method has some drawbacks. Failure to suppress reflexive saccades and loss of fixation during the test is one reason for less reliable test results (4). Also, the interpretation can be challenging as the test is prone to false positive and false negative test results (5–7), high measurement variability (8), low reliability due to learning effects (9–11), fatigue (11) and cognitive decline (12). Numerous adaptations have been proposed to improve the reliability of SAP, some of those are recent (13–16). Modifications to the testing algorithms, such as the implementation of the Swedish Interactive Testing Algorithm, primarily aimed at shortening the total test duration, did not significantly improve the test reliability (17, 18).

More recently, eye movement perimetry (EMP) has been proposed, an application that is based on the measurement of natural eye movement responses to visual features that appear at different locations in the visual field. The first EMP systems consisted of video recorded eye movement and a decision algorithm to confirm that a stimulus was detected (19). The investigators using this application visually graded all eye movements in response to stimuli with various intensity levels (range: 15 dB from dimmest to brightest stimulus). The dimmest stimuli that were seen per location were obtained to plot the visual field. In subsequent studies, EMP was combined with eye tracking technology and more objective, automated confirmation of eye movements to assess the extent of the visual fields by using primary saccades (20, 21). Good agreement was found between the visual fields assessed with EMP and SAP (20, 22). EMP combined with eye tracking also provides characteristics of the primary saccades made to the seen stimuli, such as saccadic reaction time (SRT), saccadic velocity, and fixation accuracy (21, 23). It has been shown in healthy eyes that the SRTs depend on stimulus properties e.g., size, contrast, diameter, and spatial location in the VF (22, 23) and on subject characteristics such as age (24).

Previously, we conducted several studies on saccadic properties using an adapted visual field test paradigm. In healthy eyes, we reported low variability and good repeatability of SRTs across the visual field up to 30° eccentricity (21). In patients with cataract, we found no effect of cataract severity (LOCS III grades I through IV) on SRT and the sensitivity thresholds. In patients with LOCS III grade V, however, a 27% increase in SRT compared to grades I–IV was found (25). In patients with various degrees of glaucoma, distinct differences in average SRTs were found when the same test grid as in SAP was used (22, 26). These data provided insights into global visual field characteristics. However, how visual field sensitivity loss and SRT are related is still not known. Data that combine visual field sensitivity and SRT per tested location are currently not available.

In the present study, we investigated the relation between VFS (in decibels) and SRT (in milliseconds) in corresponding locations of the visual field in patients with mild, moderate, and advance glaucoma compared to healthy controls. We were particularly interested in how the SRTs compared between locations without any sensitivity loss in glaucoma patients and the equivalent areas in healthy eyes. We hypothesized that SRTs would increase with a decrease in VFS, and that SRT would be similar between comparable locations in healthy eyes and the unaffected locations in the visual field of glaucomatous eyes, under the assumption that SAP and EMP results are similar.

## Materials and Methods

### Participants

We recruited healthy control subjects and subjects who were diagnosed with glaucoma from the Rotterdam Glaucoma Imaging Study (Netherlands Trial Register number: NTR1195) initiated by the Rotterdam Eye Hospital, Rotterdam, The Netherlands. The Rotterdam Glaucoma Imaging Study is a longitudinal study to assess established and experimental diagnostic technologies for glaucoma diagnosis. All subjects were over the age of 50 years, familiar with SAP, and naïve to the purpose of this study. Glaucoma patients with either primary open angle or angle closure glaucoma who had glaucomatous optic disc changes and corresponding visual field defects on HFA were included. Subjects with ocular or systemic disorders with known effects on the visual acuity or the visual field other than glaucoma were excluded. Participants consent was obtained before testing. The Medical Ethics Committee of the Erasmus University Medical Center, Rotterdam, The Netherlands (METC-2009-199) approved the experimental procedures. The study adhered to the Declaration of Helsinki for research involving human subjects.

### SAP and EMP Recordings

Subjects underwent a standard white on white automated visual field test (Humphrey Field Analyzer IIi, 24-2 SITA-Standard, Carl Zeiss Meditec, Dublin, CA, USA). This device tests threshold light sensitivity (dB) on a 54-location grid with the test locations evenly spaced 6 degrees apart on 8 eccentricities: at 4.2°, 9.5°, 12.7°, 15.3°, 17.5°, 21.2°, 22.9°, and 27.2°. The results of this test were summarized in the ‘total deviation plot' in which the local sensitivity deviation with respect to the age-specific normal population's sensitivity is presented for each tested location. A subject was allocated to one of 3 ‘glaucoma severity groups' or the control group. The glaucoma severity was based on the average of all local sensitivity deviations per VF into one mean deviation (MD) value. The different glaucoma groups were defined as mild glaucoma (MD above −6 dB), moderate glaucoma (MD between −6 and −12 dB), and advanced glaucoma (MD below −12 dB). Reliability criteria were as recommended by the instrument's algorithm (fixation losses <20%, false positive and false negative errors <33%). Next, each subject underwent an EMP measurement. The eye tracking setup consisted of an ‘in-plane-switching' TFT monitor (ProLite XB2776QS, iiyama, Chuo, Tokio, Japan) to which a 60 Hz eye tracker (Tobii X2-wide, Tobii Technology AB, Danderyd, Sweden) was mounted according to product specifications to follow a subject's gaze. To minimize the variability in head movements, each subject was asked to place the head on a chin rest at a 55 cm distance from the monitor. The manufacturer of the Tobii X2 reported a system's latency of <35 ms, and an accuracy of gaze tracking better than 0.5°. After completing the standard 9-point calibration, a stimulus sequence in which the stimuli were presented in random order was shown on the TFT monitor with Tobii StudioTM software running on a Dell M6800 laptop computer. A correction glass was placed in front of the tested eye to correct the spherical equivalent of any individual's refractive error, and the contralateral eye was covered with a black polymethyl methacrylate (PMMA) disc. This disc ensured monocular viewing by blocking the vision of the covered eye while it allowed the eye tracker to track both eyes. The two eyes were tested in random order. The background luminance in the trial room was continuously kept low but not completely dark, and auditory noise levels were kept to a minimum to avoid any distraction. Before each exam, a standardized instruction was given: “First fixate the dot at the center of the screen; when an extra dot is seen, simply look at it, then fixate the central dot again.”

### Test Procedure

**Figure 1:**
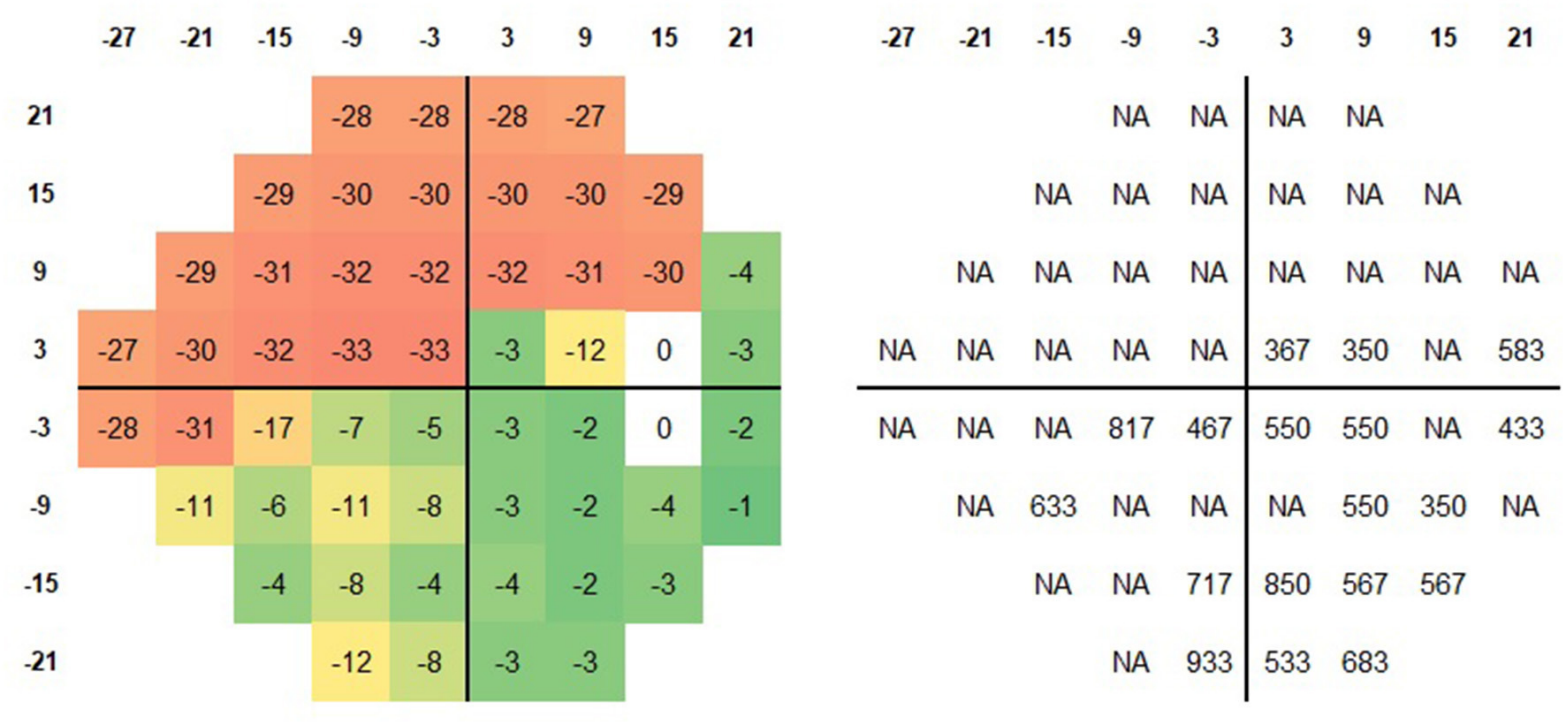
Schematic representation of the 24-2 test grid. The local sensitivity deviation values **(Left)** and the corresponding SRT **(Right)** for the brightest stimuli for a patient with advanced glaucoma (MD −18 dB) are plotted. In the majority of locations with incomplete sensitivity loss (green and yellow squares), an SRT was calculated.

In the center of the monitor, a central stimulus remained lit during the entire exam, and the background luminance was kept at 60% (126 cd/m^2^). The peripheral stimuli (equivalent Goldman size III) were presented on the monitor at four different brightness levels on a gray to white scale; 70% (162 cd/m^2^), 80% (202 cd/m^2^), 90% (244 cd/m^2^), and 100% (289 cd/m^2^). The peripheral stimulus size was corrected for eccentricity to minimize the effect of the flat monitor surface. The stimulus grid was identical to the 54 locations of the 24-4 SAP grid, allowing a pointwise comparison between SAP and EMP. Figure 1 shows a schematic representation of the used test grid. As a result, 216 unique stimuli were presented on the screen during each exam. To limit the total duration, we implemented an interactive testing paradigm. Each stimulus was projected with a maximum duration of 1,200 ms. However, once the detection algorithm confirmed the saccade toward a peripheral stimulus, the stimulus remained lit for an additional 80 ms before it disappeared. The delay between two consecutive trials was 0.2 s, thus the time between disappearance of the peripheral target and the start of a random foretime before a new peripheral target was shown. This random foretime ranged between 1.5 and 2.5 s. This algorithm reduced the testing time compared to our previous studies in which all stimuli were presented for 1,200 ms (21, 26). The total examination duration per eye was 4–5 min, whereas before, without the interactive paradigm, the test typically took 11 min.

### Data Analysis

Eye movement data were visually inspected and analyzed utilizing self-written Matlab (MathWorks, Natick, MA, USA) software (21). A trained investigator (GT) visually checked all trials to ensure validity. Further data processing was based on an earlier described method (25). In summary: an eccentricity dependent circular area around the peripheral stimuli was defined as the area of interest (AOI). The AOI radius increased linearly from 2° at the central locations to a 5-degree radius at 30° of eccentricity, because of the increase in saccade inaccuracy and imprecision with increasing eccentricity (27). Based on gaze angle data, a stimulus was classified as ‘seen,' ‘not seen,' or ‘invalid.' Of each ‘seen' stimulus, the SRT was calculated as the time difference between stimulus presentation and the onset of the saccadic eye movement. This onset was defined as the moment that gaze velocity exceeded a 50°/s threshold value. A peripheral stimulus was labeled as ‘unseen' when no eye movements were made toward the peripheral target. A peripheral stimulus was invalid when 1 the peripheral stimulus was seen but no valid SRT could be determined from the data due to blinking, 2. when the primary saccade did not land in the target area, 3. when the direction of the primary saccade was not in the direction of the peripheral stimulus or 4. when no eye movement data were available due to eye tracking failure. For a visual representation of these methods, we refer to previous work (25).

### Statistics

In each tested location, we obtained the sensitivity threshold and the SRT value. We defined 6 so-called ‘SAP sensitivity bins' to which a location was allocated with its corresponding SRT value. These bins were defined based on the total deviation as follows: bin 1: > 0 dB, bin 2: 0 till −3 dB, bin 3: −3 till −6 dB, bin 4: −6 till −12 dB, bin 5: −12 till −18 dB, and finally bin 6: −18 dB and lower. Thus, each bin contained the SRT values of visual field locations with similar sensitivity loss. A non-parametric test (Wilcoxon signed-rank test) was used to test any between-group differences. Because of the hierarchical data structure (described below), a multilevel mixed model was used (generalized linear mixed model, SPSS, IBM) to determine the effect of different factors on the dependent variable SRT. This linear regression model took both the within subject and between subject variability into account by allowing a leveled structure. Three levels were used: (1) subject, (2) eye (control, mild, moderate, advanced glaucoma), and (3) stimulus eccentricity (8 eccentricities). The 8 eccentricity levels were defined as: 4.2°, 9.5°, 12.7°, 15.3°, 17.5°, 21.2°, 22.9°, and 27.2°. The model included the following individual factors: the subject's age as a continuous variable and their gender, the stimulus intensity and the glaucoma severity (either ‘glaucoma severity group' or ‘SAP sensitivity bin,' as these are correlated) as categorical variables. Any differences between the levels within individual factors were tested with pairwise contrast estimates. This paired approach allowed us to estimate and compare the mean SRT in control eyes and eyes with mild, moderate, and advanced glaucoma. All tests were done with IBM SPSS Statistics for Windows, version 23.0 (IBM Corp., Armonk, NY), and the significance level was set at *p* = 0.05.

## Results

We included 58 control eyes of 34 subjects and 76 glaucomatous eyes of 42 glaucoma patients. Nine (9) eyes of 9 control subjects were excluded because they did not meet the inclusion criteria. Six (6) eyes of glaucoma subjects were excluded because their glaucoma was only diagnosed in the contralateral eye. Three more eyes (1 control eye and 2 glaucomatous eyes) were excluded because eye tracking failed. Of all the presented 216 stimuli per subject, a total of 67% (in controls) and 35% (in patients) were marked as seen and 10% (in controls) and 38% (in patients) were marked as unseen. The remaining points were marked as ‘invalid' due to seen peripheral target but no valid SRT (due to blinking) (8 vs. 11%), peripheral saccade not in the target area (6 vs. 4%), primary saccade in the wrong direction (4 vs. 6%), or eye tracking failure (5 vs. 6%). Both eyes were included in 24 control subjects and 34 glaucoma patients. Based on SAP mean deviation, we had 32 mild glaucoma eyes, 15 moderate glaucoma eyes, and 29 eyes with advanced glaucoma (*p* < 0.001). There was no statistically significant difference in age between the groups (*p* = 0.41). The percentage of women in the groups ranged from 33% in the mild glaucoma group to 55% in the control group (*p* = 0.19). Table 1 summarizes the demographic characteristics of all groups.

**Table 1:** Demographic characteristics of the included eyes.

	**Control**	**Mild**	**Moderate**	**Advanced**	*p*-value^*^
Number of eyes	58	32	15	29	–
Gender, % women	55.2	34.4	33.3	41.4	0.185
Age in years (range)	65.5 (50.9–85.1)	66.5 (51.9–79.8)	67.9 (51.9–79.8)	68.3 (52.9–81.5)	0.406
SAP Mean deviation dB (range)	0.2 (−1.6 to 2.6)	−2.5 (−5.7 to 1.4)	−9.6 (−11.6 to −6.9)	−17.6 (−25.8 to −12.36)	<0.001

* *Kruskal Wallis Test*.

**Figure 2:**
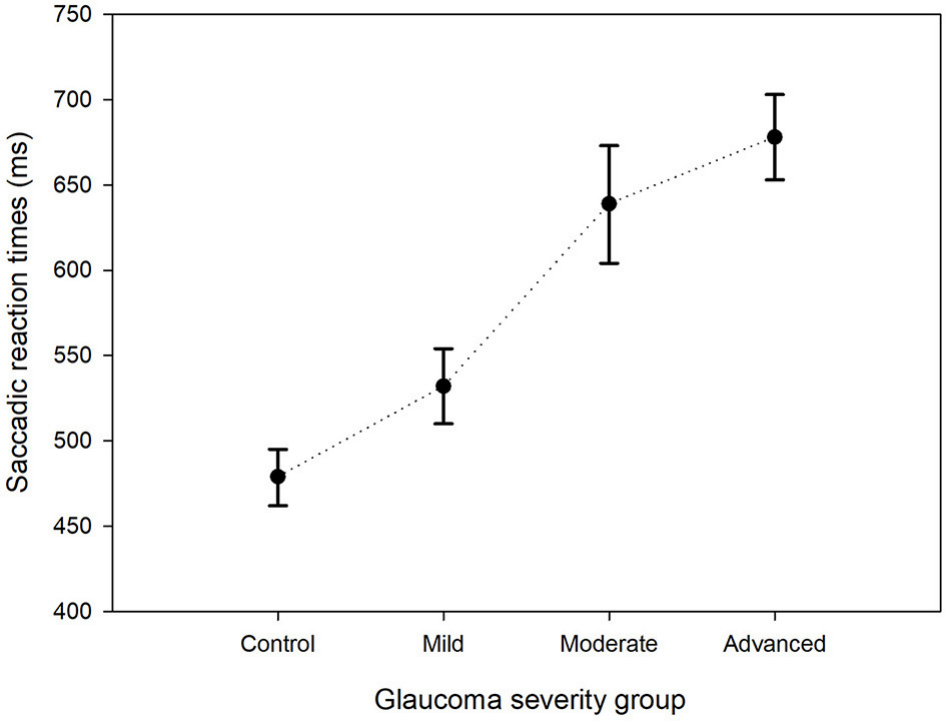
Estimated mean saccadic reaction times per group. The estimated mean SRTs and their corresponding 95% confidence intervals for the control group and the three glaucoma groups are shown.

**Figure 3:**
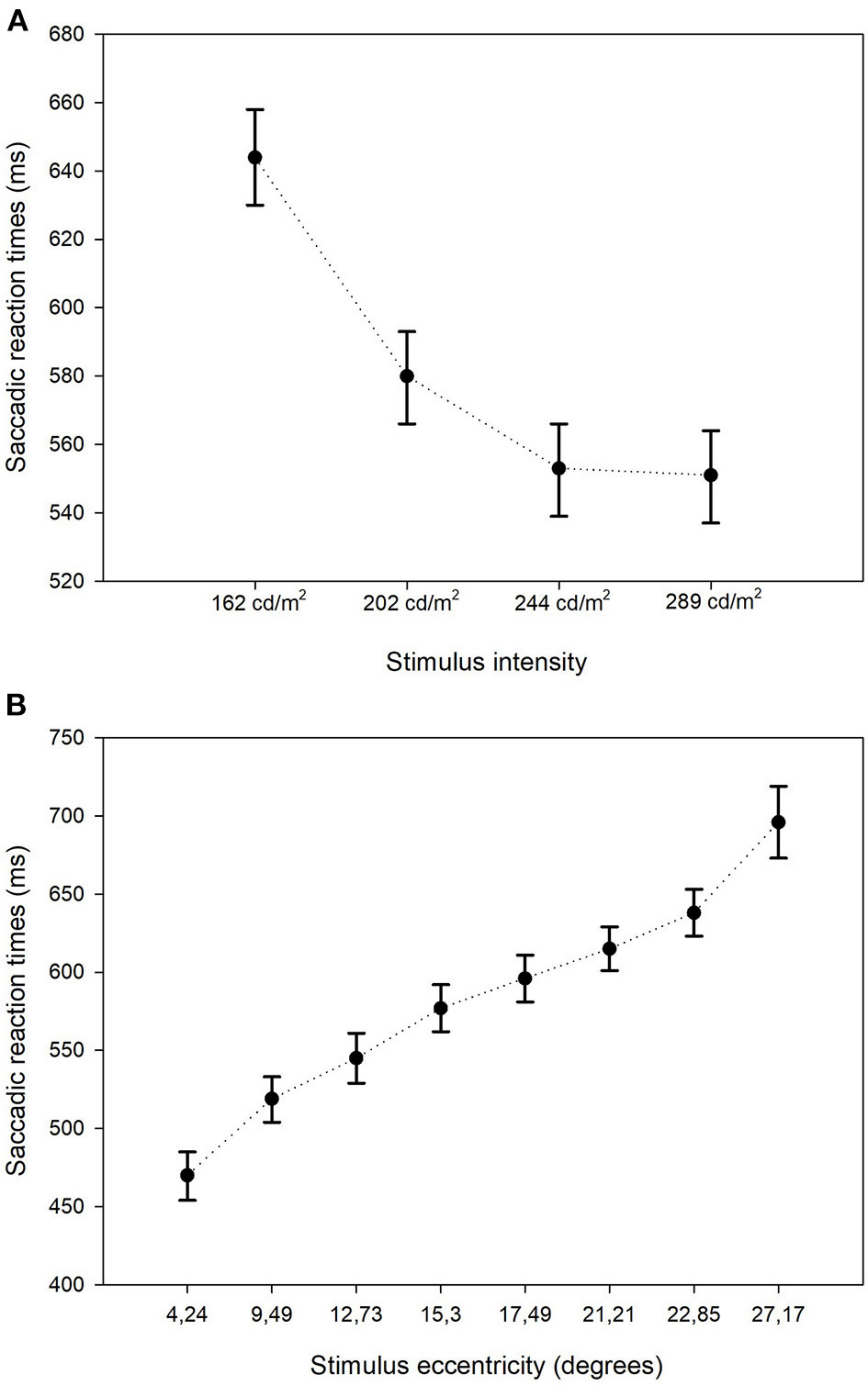
Estimated mean saccadic reactions times: stimulus intensity and eccentricity. Estimated mean SRTs and their corresponding 95% confidence intervals stratified by stimulus intensity **(A)** and by stimulus eccentricity **(B)**.

The GLMM showed that the estimated mean SRT in the better eyes (566 ms) of glaucoma patients was significantly shorter compared to the worst affected eyes (652 ms; *p* < 0.001). There was no left (476 ms) vs. right (475 ms) difference in SRT in control eyes (*p* = 0.93). The estimated mean SRT increased with increasing glaucoma severity. While in the control eyes the SRT was 479 ms, it was 678 ms in the eyes of patients with advanced glaucoma (*p* < 0.001). Figure 2 shows this relationship between SRT and glaucoma severity. Here, the estimated means and their 95% confidence intervals of all four groups have been plotted. Pairwise contrasts showed statistically significant differences between all groups (*p* < 0.001) except between moderate and advanced glaucoma. Next, we found that with dimmer stimuli and larger stimulus eccentricity, the SRT increased significantly (Figures 3A,B). At 167 cd/m^2^ (the dimmest stimulus), the mean estimated SRT was 644 ms compared to 551 ms at 289 cd/m^2^ (the brightest stimulus; *p* < 0.001). Pairwise contrasts showed statistically significant differences between the groups (*p* < 0.001), except between 244 and 289 cd/m^2^ intensity (*p* = 0.61). For the brightest stimuli, the mean estimated SRTs ranged between 470 ms [at eccentricity group I (4.2°)] and 696 ms (at eccentricity group VIII (27.2°); *p* < 0.001). In the central visual field of healthy controls, we found a similar increase of SRT with increasing eccentricity; 373 ms at 4.2°, 411 ms at 9.5°, 434 ms at 12.7°, and 467 ms at 15.3°. Pairwise comparisons revealed that there was no significant difference in SRTs between 90 and 100% stimulus intensity (*p* = 0.608) and between moderate glaucoma and advanced glaucoma (*p* = 0.063). Age (*p* = 0.072), gender (*p* = 0.89), and right/left eye (*p* = 0.38) did not statistically significantly correlate with SRT. In Appendix 1, a comprehensive overview has been listed of all factors and their levels.

### SRT vs. SAP Sensitivity Loss

**Figure 4:**
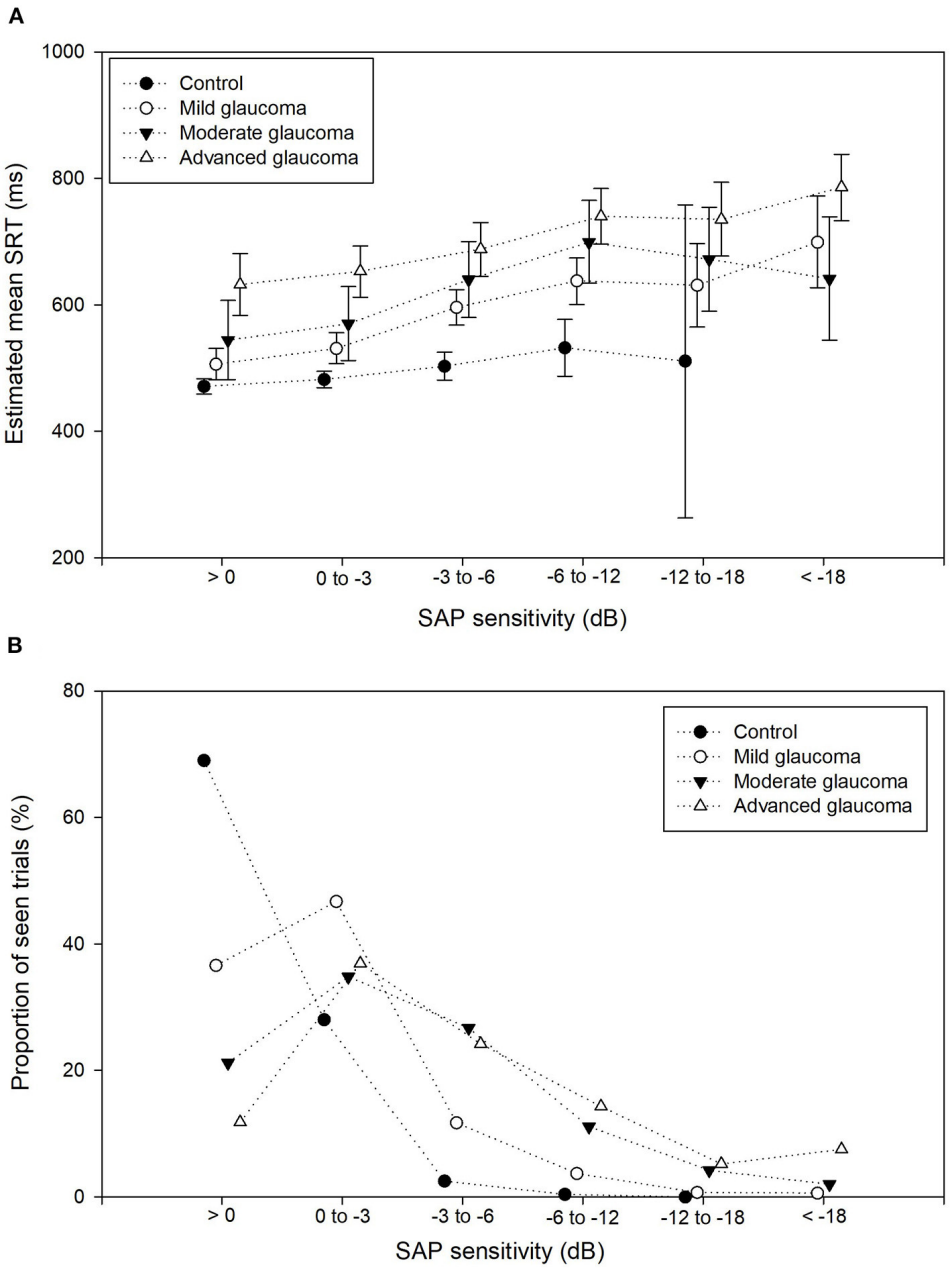
Proportion seen trials and mean SRT per sensitivity bin. **(A)** Proportion of seen trials (%) for each of the 6 *SAP sensitivity bins*, stratified by the control group and glaucoma severity group. The proportion of seen stimuli decreased drastically with increasing SAP sensitivity loss. **(B)** Estimated mean SRTs and their corresponding 95% confidence intervals for the 6 *SAP sensitivity bins*, stratified by the control group and glaucoma severity group (dotted lines) show the increase of SRT with decreased retinal sensitivity.

The percentage of seen stimuli resulting in an SRT value was on average 35% in the patient group. This percentage decreased with increasing glaucoma severity, i.e., 51, 24, and 20% in the mild, moderate, and advanced glaucoma groups, respectively. In Figure 4A, the proportion of seen stimuli with a valid SRT has been plotted against the corresponding SAP sensitivity bin and stratified by glaucoma severity. In the control group, the proportion of seen stimuli decreased significantly with increasing SAP sensitivity loss. As expected, the proportion of trials in the higher SAP sensitivity bins increased with glaucoma severity. In Figure 4B, the corresponding SRT values have been plotted for the SAP sensitivity bins, again stratified by glaucoma severity. These values represent the estimated mean SRTs and their estimated corresponding 95% confidence intervals. Here, SRT increased with increasing glaucoma severity. A clear difference in SRT values was found between each of the glaucoma severity groups up to bin 5 (−12 to −18 dB). In each glaucoma group, the SRT gradually increased with lower sensitivity. All the comparisons between the SAP sensitivity bins have been listed in Appendix 2.

**Table 2:** Between glaucoma severity groups comparisons for each of the 6 *SAP sensitivity bins*.

**Compared groups**	**Bin 1**	**Bin 2**	**Bin 3**	**Bin 4**	**Bin 5**	**Bin 6**
	***p*** **-value**					
Control—mild	**0.049**	**0.002**	** <0.001**	0.342	0.527	–
Control—moderate	** <0.001**	** <0.001**	** <0.001**	**0.015**	0.440	–
Control—advanced	** <0.001**	** <0.001**	** <0.001**	**0.014**	0.395	–
Mild—moderate	**0.024**	**0.001**	**0.001**	**0.019**	0.609	0.147
Mild—advanced	** <0.001**	** <0.001**	**0.001**	**0.010**	0.449	0.978
Moderate—advanced	0.109	0.115	0.729	0.846	0.854	0.121

*Statistically significant differences (p < 0.05) in bold letterpress*.

The largest difference in SRT was found between the control group and the advanced glaucoma group: 161 ms, 171 ms, 185 ms (*p* < 0.001), 208 ms (*p* = 0.014), and 224 ms (*p* = 0.40) in bins 1, 2, 3, 4, and 5, respectively. Only one seen stimulus was included in the control eyes in bin 5 (see Figure 4A) compared to an average of 41 seen stimuli in the glaucoma groups in bin 5. This resulted in an unreliable increase of the corresponding 95% confidence intervals of the estimated mean. In Table 2, the details on each of the pairwise comparisons have been listed between the glaucoma severity groups per SAP sensitivity loss. Up to bin 4, most of the differences between the glaucoma severity groups in Table 2 were statistically significant. Interestingly, this also included the difference between the control group and the moderate/advanced glaucoma groups in bin 1 and 2. In these locations with very mild VF sensitivity loss, the mean estimated SRTs were significantly increased between the three glaucoma severity groups and the control group.

## Discussion

We investigated the relationship between SAP visual field sensitivity (VFS) and EMP saccadic reaction time (SRT). We confirmed previous findings that the SRT increased with greater SAP sensitivity loss. Interestingly, we also found that at locations with normal SAP sensitivity, however, glaucomatous eyes showed a significant increase in SRT compared to healthy eyes.

### Saccadic Reaction Times in the Glaucomatous Visual Field

In our study, we primarily focused on the between location correlations rather than on the between-subjects correlations. All visual field locations with corresponding SAP sensitivity loss were grouped in one of the 6 SAP sensitivity bins. For each glaucoma severity level, the SRT values significantly increased. An overall increase in SRT between controls and advanced glaucoma was found of 42% (199 ms). Few data have been published on SRTs at locations without detectable visual field loss in patients diagnosed with glaucoma. A recent study by Najjar et al. reported hypometric and reduced velocity, amplitude, and gain in horizontal saccadic eye movements in patients with primary open-angle glaucoma without detectable glaucomatous visual field loss (28). The authors, however, did not report lengthened SRTs. In the present study, we found that within each of the SAP sensitivity bins, the estimated SRTs increased with increasing glaucoma severity. Even at locations with normal sensitivity, the SRTs showed increased values in the glaucomatous eyes.

### Pre-perimetric Glaucoma

Optical coherence tomography (OCT) can detect glaucomatous damage to retinal ganglion cells and is more sensitive than SAP in detecting early glaucoma (29, 30). OCT nerve fiber layer measurements are more sensitive to detecting early damage than optic nerve head (ONH), macular and confocal scanning laser ophthalmoscope measurements (31, 32). Characteristic glaucomatous nerve fiber layer or optic disc damage (assessed with OCT) and in fundoscopic examinations) without visual field defects (assessed in SAP) is known as pre-perimetric glaucoma. OCT performs well in detecting preperimetric glaucomatous damage and provides additional information to fundoscopy and SAP (33). It is essential to recognize early forms of glaucoma and consider ocular hypotensive therapy because early treatment significantly reduces the risk of disease progression (34), even in pre-perimetric glaucoma (35). In turn, our data suggest that the increase of SRT, as measured with EMP, detects functional glaucomatous changes before detectable SAP sensitivity loss occurs. This is supported by the fact that at least 20–50% of retinal ganglion cells are lost before associated sensitivity loss in the visual field occurs (36–38). Our findings could implicate that motor-responses such as saccades are affected earlier than the sensory perception of stimuli in the visual field. It would be interesting to do a follow-up study to investigate how SRTs change with progressive disease, either pre-perimetric or perimetric.

### Saccade Initiation and Eye Movements

Saccade programming, planning, and initiation is a highly complex process that involves numerous structures and pathways. Important nuclei are the lateral geniculate nucleus (LGN) in the basal ganglia, the superior colliculus (SC) and paramedian pontine reticular formation (PPRF) in the brain stem, and the frontal eye fields (FEF) in the frontal cortex (39). Signs of neurodegeneration (40, 41) of key brain structures in patients with open angle glaucoma and normal tension glaucoma (42, 43) have been described by a growing number of imaging studies and laboratory studies (44). What the exact causative relation is remains unclear and future studies will have to show if brain imaging can support clinical treatment of glaucoma patients. SRT is the sum of the total processing time, that consists of localizing the stimulus, planning, and initializing the saccadic eye movements. To what extent the different structures in the retina, frontal lobe, basal ganglia, and brainstem contribute to the increase of SRT in glaucoma is not clear. It has been suggested that the delayed SRT could be the result of damage to the retinal ganglion cells that project directly to the superior colliculus (SC) (45–47). Generally, the first sensitivity loss in glaucomatous visual fields occurs in the peripheral field. Walker et al. suggested that saccade generation may result from competitive interactions between the fixation system and the ‘move' system within and downstream of the SC (48). Peripheral sensitivity loss in glaucoma patients may very well favor the fixation system and have an accompanying negative effect on saccade initiation. The delay in SRT may arise in the first critical steps of visual processing in the retina, i.e., in the horizontal, bipolar, and ganglion cell layers. These cells are the first to be affected by the loss of nerve fibers (44). In recent studies that included timing of primary saccades to seen stimuli, a delay in saccade onset was found in glaucoma (26, 49). This delay was present in mild, moderate, and advanced glaucoma and increased with glaucoma severity (26, 45). Najjar et al., who studied saccades in pre-perimetric glaucoma and healthy subjects, did not find SRT differences between glaucoma patients and controls (28). This may be related to the different paradigm they used. It is also possible that the differences in our findings are caused by differences in the study populations. Also, several studies have shown that in patients with glaucoma, saccadic eye movements are affected in terms of amplitude, fixation duration, velocity, accuracy, and scan paths (28, 49–52). However, other studies did not find significant differences between glaucoma patients and healthy controls (45). Although not addressed in this study, these differences in saccade properties could suggest that the findings are dependent on the implemented test paradigm.

### Factors That Affect Saccadic Reaction Times

Altered SRTs in intact parts of the visual field have also been reported in patients with several (neurologic) conditions such as Parkinson's disease (53) and in patients with brain lesions (54, 55). Bola et al. found impaired visual processing speed in the intact regions of the visual field of patients with pre- and post-chiasmatic lesions (54). The reaction times were related to the functional state of the surrounding visual field, as well as to the size of the visual field defect. It was suggested that these deficits could also contribute to subjective vision loss and therefore influence rehabilitation (55). When reviewing the literature of SRTs in prosaccades, we see large differences between stimulus conditions. The different study protocols vary considerably and therefore interpretation of the outcome and putting this into perspective with other work is not straightforward. Some examples are the differences in stimulus to background ratios and the dimensions of the tested visual field including differences in the directions of the eye movements. Most studies were limited to a small number of locations, mostly shown on a straightforward horizontal or radial axis grid, instead of a larger visual field grid used in standard automated perimetry, as we did in our study. In the present study, we were able to confirm previously reported SRT characteristics measured with EMP. The mean SRT difference between the dimmest and brightest stimuli in this study was 93 and 226 ms between the stimuli with the smallest and largest eccentricity. The magnitude of the SRT increase was comparable to what we have reported in previous work (21, 25). However, compared to the work of other groups, the SRTs increase as a function of eccentricity was striking and the SRTs that we found were relatively long compared to other groups. In these other studies, SRTs are found to be dependent of eccentricity (56), independent of eccentricity (57–59) or dependent only in a specific eccentricity range (27, 60). For example, Fuller et al. found an increase of SRT with slopes of 0.64–1.96 ms per degree of eccentric dependent on orbital starting position of the eye (56). The white stimuli that they used were flanked by a green and a red LED light and were presented at eccentricities ranging between 10 and 60 degrees on a horizontal axis. In a study in the 70's by Frost and Pöppel, all SRT were approximately 250 ms over a range of 40° (5°-45°) (57). The presented stimuli were white and 32 cd/m^2^ in intensity, and again shown only on the horizontal meridian. Dafoe et al. implemented a grid with 8 radial directions but tested only the central 8° of the visual field (58). They concluded that SRT was longer for the inferior hemi-field compared to the superior hemi-field and longer for vertical compared to horizontal targets, but found no effect of eccentricity. One exception was the most central test location at 0.5° which was removed from the final analyses because of low reliability of the data. Similar findings are reported by Casteau and Vitu in a 0.5–6 degree horizontal grid setup in a control experiment (59). The mean SRT was ~175 ms except for most central 1° test location (195 ms). In another experiment conducted by the same group these results were reproduced and confirmed on eccentricities between 2 and 15 degrees in several directions (0–90 degree). They did find increased SRTs for stimuli presented at <2° and >15° and also showed small variations in SRT in differences in direction. Lastly, Kalesnykas and Hallet found that SRT was quite stable of the central 12°, again with an exception for foveal stimuli and more peripherally presented stimuli (60). The red and green targets, with a luminance just above the dark-adapted foveal threshold, were presented in the peripheral nasal and temporal field and showed marked increases of SRT for eccentricities over 22° and in the region of the blind spot.

In contrast to these studies that report almost no SRT increase with an increase of eccentricity in the central 1–2° to 12° range, with an exception for the smallest (foveal) and more peripheral (roughly >15°) eccentricities, we found a more gradual increase in SRT over a wide range of eccentricities from 4.2 to 27.2 degrees in healthy control and glaucomatous eyes. Even compared to the experiment of Vitu et al. with clear similarities between their and our testing paradigms in the central visual field. To our knowledge this central SRT increase has not been reported before. This marked differences with eccentricity may be more prominent in our test due the higher degree of uncertainty associated with a high number of stimulus locations. The effect of stimulus to background ratios on SRT needs to be studied in more detail before any definitive conclusions can be drawn.

### Study Limitations

As previously discussed, stimulus intensity is an important factor of SRT. The test grid in EMP and SAP was identical in this study, but the range of stimulus to background contrast ratios is considerably higher in SAP. In studies that use TFT monitors as projection screens, the contrast ratios are limited by the maximum luminance of the monitor. Despite this limitation, this approach does enable the end-user to assess differences in SRT. Higher contrast ratios could be achieved by other types of monitors or projection systems. Presumably, when lowering the stimulus to background contrast, this could lead to an increase in SRT variability. The increase of the SRT confidence intervals at greater SAP sensitivity loss (see Figure 4) suggests a reduced accuracy of the estimated mean SRT by the mixed model approach. The large confidence intervals were the result of the small number of trials that were included in these subgroups. This is, however, inherent to this type of data due to the scarcity of visual field points with very advanced visual field damage. In future experiments the intention should always be to try to limit the number of missing data, such as the missing values due to eye tracking failure. And, if testing time permits, repetition of identical trials is desirable to include seen stimuli including their SRT values. Also, SAP measurements are known to be subject to learning effects (10, 11). In this study, the subjects had already gained SAP experience. In previous work, the measurement variability in SRT between three subsequent measuring series was shown to be small in EMP (21).

### Clinical Applicability

In recent years, eye tracker-based eye movement perimetry has gained popularity. Several research groups have picked up this approach to test the size and shape of the visual field (61, 62). The use of the natural response toward newly appearing stimuli in the visual field has the potential to improve visual field testing. Additional benefits of EMP are the low risk of false-positive errors, which are a known source of unreliability in SAP (6, 7), and patients' preference of EMP over conventional SAP (63). For clinical application, it would be interesting to compare SRT values obtained in patients with age-adjusted normative values. If EMP can detect glaucoma in a latent stage of the disease, this technique could be of use for screening purposes (63, 64).

## Conclusions

This study showed that in patients with primary open angle glaucoma, saccadic reaction times, measured with eye movement perimetry, are increased in locations without detectable visual field sensitivity loss on standard automated perimetry. We speculate that this increase in SRT may result from pre-perimetric damage, i.e., structural damage not yet noticeable with SAP. This information may enable the development of tools that can quantify early functional changes in glaucoma. One essential comment is that saccadic initiation is affected by several factors, not merely by local retinal sensitivity loss. Further research will be required to investigate the underlying mechanisms and the effects of glaucoma on visual processing and saccade initiation.

## Data Availability Statement

The raw data supporting the conclusions of this article will be made available by the authors, without undue reservation.

## Ethics Statement

The studies involving human participants were reviewed and approved by Medical Ethics Committee of the Erasmus University Medical Center. The patients/participants provided their written informed consent to participate in this study.

## Author Contributions

GT performed the measurements, analyzed and interpreted the data, and was a major contributor in writing the manuscript. HL designed the study, supported patient recruitment, and interpreted the data. KV designed the study and interpreted the data. JS designed the study and interpreted the data. JP designed the study, interpreted the data, and was contributor in writing the manuscript.

## Conflict of Interest

The authors declare that the research was conducted in the absence of any commercial or financial relationships that could be construed as a potential conflict of interest.

## Publisher's Note

All claims expressed in this article are solely those of the authors and do not necessarily represent those of their affiliated organizations, or those of the publisher, the editors and the reviewers. Any product that may be evaluated in this article, or claim that may be made by its manufacturer, is not guaranteed or endorsed by the publisher.

## APPENDIX

**APPENDIX 3:** The results of the multilevel model of the individual factors (bold) and their levels.

	**Saccadic reaction time (ms)**		
	**Parameter estimate**	**95% CI**	***p*-value**
Intercept	678	583–773	** <0.001**
Age	1.2	–0.1 to 2.5	0.072
**Eye**			
Right	10	–12 to 31	0.378
Left	Reference		
**Eccentricity**			
1	–226	–247 to –205	** <0.001**
2	–177	–198 to –157	** <0.001**
3	–151	–172 to –129	** <0.001**
4	–119	–139 to –98	** <0.001**
5	–100	–120 to –79	** <0.001**
6	–81	–101 to –61	** <0.001**
7	–57	–78 to –37	** <0.001**
8	Reference	–	–
**Gender**			
Male	–2	–20 to 24	0.884
Female	Reference	–	–
**Stimulus intensity**			
70%	93	86–100	** <0.001**
80%	29	23–36	** <0.001**
90%	2	–5 to 8	0.608
100%	Reference	–	–
**Glaucoma severity**			
Control	–202	–229 to –170	** <0.001**
Mild	–146	–179 to –113	** <0.001**
Moderate	–40	–81 to 2	**0.063**
Advanced	Reference	–	–

**APPENDIX 4:** Between groups comparisons corresponding to the data presented in panel A of Figure 4.

**Compared bins**	**Control**	**Mild**	**Moderate**	**Advanced**
	***p*** **-value**			
1 and 2	**0.002**	** <0.001**	0.168	0.267
1 and 3	**0.001**	** <0.001**	** <0.001**	**0.007**
1 and 4	**0.007**	** <0.001**	** <0.001**	** <0.001**
1 and 5	0.753	** <0.001**	** <0.001**	**0.001**
1 and 6	**–**	** <0.001**	0.054	** <0.001**
2 and 3	**0.028**	** <0.001**	** <0.001**	**0.015**
2 and 4	**0.026**	** <0.001**	** <0.001**	** <0.001**
2 and 5	0.822	**0.002**	**0.002**	**0.002**
2 and 6	**–**	** <0.001**	0.145	** <0.001**
3 and 4	0.227	**0.009**	**0.008**	**0.004**
3 and 5	0.953	0.275	0.343	0.075
3 and 6	**–**	**0.004**	0.981	** <0.001**
4 and 5	0.869	0.844	0.447	0.874
4 and 6	**–**	0.101	0.259	**0.049**
5 and 6	**–**	0.147	0.582	0.082

*Statistically significant differences (p < 0.05) in bold letterpress*.
